# Recent advances in the use of high flow nasal oxygen therapies

**DOI:** 10.3389/fmed.2022.1017965

**Published:** 2022-10-10

**Authors:** Kara D. Wyatt, Neha N. Goel, Jessica S. Whittle

**Affiliations:** ^1^Scientific Consultant, Chattanooga, TN, United States; ^2^Division of Pulmonary, Critical Care, and Sleep Medicine, Department of Medicine, Icahn School of Medicine at Mount Sinai, New York, NY, United States; ^3^Department of Emergency Medicine, University of Tennessee, Chattanooga, TN, United States; ^4^Vapotherm, Inc., Exeter, NH, United States

**Keywords:** high-velocity therapy, high-flow oxygen, respiratory failure, non-invasive ventilation, high flow nasal cannula, respiratory distress

## Abstract

High flow nasal oxygen is a relatively new option for treating patients with respiratory failure, which decreases work of breathing, improves tidal volume, and modestly increases positive end expiratory pressure. Despite well-described physiologic benefits, the clinical impact of high flow nasal oxygen is still under investigation. In this article, we review the most recent findings on the clinical efficacy of high flow nasal oxygen in Type I, II, III, and IV respiratory failure within adult and pediatric patients. Additionally, we discuss studies across clinical settings, including emergency departments, intensive care units, outpatient, and procedural settings.

## Introduction

High flow nasal oxygen (HFNO) is a relatively new modality for treating patients with respiratory failure. Historically, the term 'high-flow' referred to an increased bore size of the nasal cannula with associated gas flow. Technological advancements have significantly augmented this concept creating a class of devices called “high flow nasal oxygen.” While there are a variety of HFNO machines available, they can broadly be divided into two groups. Classic HFNO utilizes a high-flow nasal cannula providing heated, humidified air at flow rates up to 60 Liters/min with a corresponding fraction of inspired oxygen from 21 to 100%. The second category is high-velocity nasal insufflation (HVNI), which utilizes small-bore nasal cannulas to flush large airways, reducing anatomic dead space and increasing oxygen. Flow levels are limited to 40 L/min, but the air has greater kinetic energy resulting in a larger flush at equivalent flow rates (1). This flush difference led to different FDA classifications (DEN170001). Whether these engineering differences have a measurable clinical impact remains a subject of study.

The physiological benefits of HFNO are well-described, including decreased work of breathing, improved tidal volumes, modest increases in positive end-expiratory pressure, enhanced mucociliary clearance of secretions, and accurate delivery of FiO_2_ (2–5). Mechanistically, HFNO has many beneficial characteristics, but clinical efficacy is debated. For this review, an electronic literature search was conducted using PubMed and Google Scholar to summarize recent findings within key adult and pediatric patient populations.

## Clinical efficacy of high flow nasal oxygenation

### Type I respiratory failure

#### Adult patients

Acute hypoxemic respiratory failure (AHRF) describes patients with inadequate tissue oxygenation associated with partial pressures of oxygen < 60 mmHG. Non-invasive positive pressure ventilation (NIPPV) is associated with improved oxygenation but may cause lung damage through overdistention (6). HFNO increases alveolar recruitment relative to conventional oxygen therapy (COT) without negatively affecting tidal volume (6) and improves oxygenation, inspiratory effort, respiratory rate (RR), lung volume, and other metrics compared to face masks (7). These benefits led to recommendations for HFNO over NIPPV for treatment of AHRF by the American College of Physicians (8) and a strong recommendation with moderate certainty using GRADE guidelines for usage over COT during hypoxemic respiratory failure by a joint panel of experts within the European Society of Intensive Care Medicine (9). Moreover, HFNO may provide some advantages in terms of patients' comfort compared to NIPPV, and it is generally considered a well-tolerated device despite few studies having specifically addressed this aspect (10). However, it has been shown that higher temperature may negatively impact comfort regardless of flow rate and in more severe patients, higher flow rates may improve comfort (11). Therefore, HFNO settings that produce both optimal comfort and therapeutic effect vary with individual patients.

#### Adult critically ill patients in the intensive care unit

One of the significant recent studies was the FLORALI trial which compared intubation rates in 310 adult ICU patients with AHRF (Table 1). The authors reported no difference in intubation rates at 28 days with HFNO usage. However, there were significantly higher in-ICU mortality and 90-day mortality rates with COT and NIPPV compared to HFNO (12). In contrast, HFNO-treated AHRF ICU patients had significantly lower intubation rates than COT and significant improvements in PaCO_2_, PaO_2_/FiO_2_, and RR (14). Unlike the FLORALI study, there was no significant difference in mortality.

**Table 1:** Overview of key clinical trials and studies.

**HFNO usage**	**Authors**	**Trial name/ study design**	**Sample/participants**	**Main findings**
Acute hypoxemic respiratory failure (AHRF)	Frat et al. (12)	FLORALI Multicenter, randomized open-label trial	310 adult ICU patients	• No statistically significant difference in 28-day intubation rate between HFNO (38%), NIV (50%), and COT (47%). • Significantly higher in-ICU mortality and 90-day mortality in COT and NIV.
	Azoulay et al. (13)	HIGH – RCT Multicenter, parallel-group RCT	776 adult immunocompromised ICU patients	• No significant difference in 28 or 90-day mortality between HFNO and COT. • No significant difference in the incidence of invasive mechanical ventilation (IMV) between HFNO (38.7%) and COT (43.8%). • No difference in comfort, dyspnea scores, ICU, or hospital length of stay.
	Andino et al. (14)	Open-label, controlled, and single-center clinical trial	46 ICU patients	• HFNO-treated patients required significantly less intubations (33%) than COT (63%). • HFNO-treated patients had significantly improved PaO2/FiO2, PaCO2, and RR over COT. • No significant difference in mortality between HFNO-treated (25%) and COT (18%). • No significant difference in subjective comfort scales reported between groups.
	Coudroy et al. (15)	FLORALI-IM Multicenter, open-label RCT	300 adult immunocompromised ICU patients	• No significant difference in 28-day mortality rate between HFNO alone (36%) and alternating NIV with HFNO (35%). • No significant difference in ICU mortality rate at 90 or 180-days between HFNO or NIV. • No difference in length of ICU and hospital stay at day 28.
AHRF–emergency department	Jones et al. (16)	HOT-ER Single-center, pragmatic, open prospective RCT	303 patients with acute hypoxia	• No difference in HFNO or COT required NIV or IMV. • No difference in ED length of stay with HFNO (4.5 h) and COT (4.9 h) or hospital length of stay with HFNO (5 days) and (5.6 days) COT. • No difference between HFNO (9.1%) and COT (8.0%) in-hospital mortality.
	Macé et al. (17)	Bi-center, Prospective before-after study	102 patients in respiratory failure	• HFNO-treated patients showed significant improvement in respiratory failure signs (61%) vs. COT (15%). • Significantly more patients reported improved dyspnea with HFNO (92%) than COT (56%).
COVID-19	Perkins et al. (18)	RECOVERY-RS Parallel group, open-label, adaptive, 3-group, RCT	1,273 patients with AHRF and COVID-19	• Significantly less tracheal intubations or mortality within 30 days occurred in CPAP-treated patients (36.3%) than within COT-treated patients (44.4%). • There was no difference in tracheal intubations or mortality within 30 days in HFNO-treated patients (44.3%) or COT-treated patients (45.1%).
	Crimi et al. (19)	COVID-HIGH Open-label, parallel-group RCT	364 patients with COVID-19 pneumonia and mild hypoxemia	• No significant difference in escalation of respiratory support between HFNO (30.3%) and COT (38.6%). • No significant difference in clinical recovery between HFNO (69.1%) and COT (60.8%). • No significant difference between 28 and 60-day mortality. COPD	Lee et al. (20)	Prospective, observational study	92 AECOPD patients	• No significant difference in intubation rate between NIV (27.3%) and HFNO (25%) at day 30. • No significant difference in 30-day mortality between NIV (18.2%) and HFNO (15.9%). • pH, PaO2, and PaCO2 were not significantly different at 6 or 24 h between HFNO and NIV.
	Crimi et al. (21)	Prospective, observational study	15 Patients with AECOPD and bronchiectasis	• Patients treated with HFNO over 72 h had significant improvements in RR, pCO2, pO2, Borg score, mucus production, and subjective ease of expectoration.
Post-operative respiratory failure	Stéphan et al. (22)	BIPOP study Multicenter, randomized, non-inferiority trial	830 cardiothoracic surgery patients	• HFNO was not inferior to BIPAP; treatment failure occurred in 21.9% of BiPAP patients and 21.0% of HFNO. • Reintubation occurred in 13.7% of BiPAP patients and 14.0% of HFNO-treated patients.
Type IV Respiratory Failure	Mauri et al. (23)	Explorative physiologic study	25 non-intubated patients with extrapulmonary sepsis or septic shock	• Respiratory effort and drive were significantly improved with HFNO in comparison to LFO.
Procedures	Frat et al. (24)	FLORALI-2 Multicenter, non-blinded, open-label, parallel-group RCT	313 patients preoxygenated prior to intubation for acute hypoxemic respiratory failure	• No significant difference in patients with severe hypoxemia after preoxygenation with NIV (23%) or HFNO (27%).
	Nay et al. (25)	ODEPHI trial Multicenter, open-label, assessor-blinded RCT	380 gastrointestinal endoscopy patients	• HFNO significantly decreased the rate of hypoxemia SpO2 ≤ 92% compared to COT.
	Thiruvenkatarajan et al. (26)	OTHER (26) Multicenter RCT	132 patients undergoing procedural sedation	• No significant difference in hypoxemic events in HFNO (7.7%) or COT with mouthguard patients (9.1%). • No significant difference between HFNO or COT in requirement for interventions.

The data are less clear in critically ill, immunocompromised patients. In a study of 776 such patients with AHRF, HFNO was not superior to COT in reducing 28-day mortality (13). Other studies in this population found no difference between HFNO and venturi mask in escalation to intubation or NIPPV (27). Given sample size differences, the studies may have been underpowered to detect a difference in efficacy between HFNO and COT. In a retrospective study, immunocompromised patients treated with HFNO or NIPPV showed significantly lower intubation rates and mortality with HFNO (28). However, the authors noted significant differences at baseline, patients with more severe illnesses were treated with NIPPV and not evenly distributed between interventions (28). Therefore, these results may be an outlier among more extensive studies. The recent FLORALI-IM study compared mortality rates in severely immunocompromised patients treated with alternating NIPPV and HFNO or HFNO alone. Consistent with prior studies, the authors found no significant difference in mortality, intubation rates, length of stay (LOS) in the ICU and hospital, and ventilator-free days (15, 29).

#### Adult patients in the emergency department

Mace et al., 2019 compared oxygen treatments in 102 ED patients with acute hypoxemia. 61% of HFNO patients showed improvement in respiratory failure symptoms after 1 h compared to 15% improvement in COT-treated (17). However, there was no significant difference in LOS within the ED or intubation rates (17). Similarly, the HOT-ER study (*n* = 303) found no difference in the need for mechanical ventilation in the ED, LOS in the ED or hospital, and no difference in mortality in patients with respiratory distress (16).

In 204 ED patients, HVNI was non-inferior to NIPPV for all-cause respiratory distress in patients without a need for emergency intubation (30). In adult patients with AHRF, HVNI produces similar intubation rates and clinical outcomes. These studies also demonstrated the safety of HFNO and HVNI usage outside the context of the ICU.

#### Neonates/pediatric patients

Respiratory support for preterm infants and young children in respiratory failure can be provided through non-invasive methods prior to endotracheal intubation. Recent studies have sought to evaluate the efficacy of HFNO compared to non-invasive options to ensure clinical outcomes are not worse than standard practices. Unfortunately, many studies provide contradictory evidence, which may be due to variability of methods, flow rates, and patient populations. Recent meta-analyses suggest HFNO has a higher risk of treatment failure in infants (31–33). In a subgroup of infants (<2 yo) by diagnosis, the significant increase in HFNO treatment failure risk was specific to patients diagnosed with acute viral bronchiolitis (31).

Individual studies have found HFNO non-inferior to nCPAP or BiPAP (12), with no significant differences in treatment failure or intubation rates in preterm or pediatric patients (34–36). Non-invasive methods (nCPAP) are associated with nasal injury, particularly in infants (37). Preterm or low-weight newborns are at higher risk of skin breakdown and injury from nCPAP, resulting in higher pain scores and salivary cortisol concentrations than HFNO (38). Therefore, given the protective benefits of HFNO, more RCTs are needed to identify specific pediatric patient populations that receive the most clinical benefit.

#### COVID-19 respiratory failure

COVID-19 infections resulted in critically-ill patients with AHRF worldwide (39). In many cases, invasive mechanical ventilation was required due to the clinical severity. Initially, concerns about the risk of HFNO spreading COVID-19 through aerosolization were expressed. However, recent evidence confirmed that HFNO poses a low risk of spread, and delivery with a surgical mask prevents aerosol dispersal (40–43).

Recent studies have evaluated whether HFNO has a specific benefit over COT during COVID-19. HFNO-treated adults with COVID-19 had significantly reduced need for intubation (44, 45) and reduced time to recovery (44). Despite reduced intubation risk, HFNO was not associated with decreased LOS in the hospital or ICU, and mortality rates were unchanged (44, 45). HFNO for COVID-19-associated hypoxemic respiratory failure may reduce the likelihood of intubation, but the effect on recovery time is unclear. The RECOVERY-RS trial compared CPAP or HFNO to COT in patients with COVID-19-related respiratory failure (*n* = 1,273). CPAP reduced intubation risk and mortality over COT, but HFNO did not (18). This trial stopped early due to waning COVID-19 numbers; therefore, the HFNO group might have been underpowered to detect the same benefit. In COVID-19-pneumonia patients with mild hypoxemia (*n* = 364) there was no significant difference in escalation to respiratory support (30.3% HFNO, 38.6% COT) or clinical recovery (69.9% HFNO, 60.8% COT) (19). The authors report that this trial may have been underpowered to detect the hypothesized difference between the groups due to a lower event rate than anticipated (19).

### Type II respiratory failure

#### Acute hypercapnic respiratory failure in adult patients

Acute respiratory failure with hypercapnia is common in chronic obstructive pulmonary disease (COPD) patients, and NIV is the current gold standard for care (46). Given the physiological benefits, ease of use, and comfort, it has been increasingly studied in COPD patients and those with hypercapnic respiratory failure (47). Hypercapnic respiratory failure treatment success with HFNO suggests that patients have some assistance with ventilation and oxygenation.

In patients with severe COPD and ventilatory limitations during exercise, HFNO improved endurance time and dyspnea rates over COT (48). Additionally, HFNO enhanced ventilation efficiency in COPD patients (49, 50). In 19 COPD patients, HFNO improved pCO_2_ despite reduced calculated minute ventilation, suggesting a reduction in dead space ventilation (51). One hundred two severe COPD patients with hypercapnia had no difference in pCO_2_, quality of life, or dyspnea symptoms after 6 weeks of HFNO or NIPPV usage (52). Additionally, increased mean airway pressures have been reported in COPD patients (53). In 14 stable, severe COPD patients on HFNO, COT, or NIPPV, HFNO patients showed reduced inspiratory effort, RR, and pCO_2_ compared to COT (54). For severe COPD patients with intrinsic PEEP, vulnerable to dynamic hyperinflation, the smaller amount of positive pressure generated by HFNO compared to NIPPV could be beneficial.

In the acute care setting, NIPPV has significant benefits and remains strongly recommended for acute exacerbation of COPD (AECOPD) (55, 56). However, NIPPV remains underutilized and, when utilized, fails in 10–25% of patients (57–59). Evidence for using HFNO in outpatient settings for stable COPD is promising, but its role during AECOPD is still unknown. Given its ease of use and tolerability, HFNO utilization is increasing in all acute care settings for all causes of respiratory failure. In 15 patients with AECOPD and bronchiectasis, HFNO usage improved dyspnea, gas exchange, mucus production, and decreased RR without any significant safety concerns (21). A study of 92 patients with AECOPD showed no difference in intubation rate or mortality with HFNO compared to NIPPV (20). Several ED studies comparing HFNO to NIPPV or COT for mixed or undifferentiated respiratory failure showed no difference in outcomes between the different modalities, with one showing HFNO to be non-inferior to NIPPV (16, 30, 60, 61). In a recent case study, an elderly male with severe bronchiectasis was prescribed HFNO for long-term home usage and displayed reduced mucus buildup after 6 months (62). More studies are needed to delineate the benefits of HFNO home usage.

HVNI was shown to have comparable efficacy to NIPPV in hypercapnic respiratory failure, with no significant difference in treatment failure or intubation rate (63). Multiple studies confirm that HFNO produces similar treatment failure, intubation, and mortality rates (20, 63–65) and provides similar RR, PaCO_2_, and PaO_2_/FiO_2_ ratios (64, 66). Evidence for HFNO usage during COPD with hypercapnia is growing, but more studies are needed.

#### Hypercapnia in pediatric patients

Hypercapnia is relatively rare in children and is typically associated with advanced lung diseases, such as cystic fibrosis or neuromuscular diseases (67–69). Affected children may require NIV to offset the effects of nocturnal hypercapnia to reduce the risk of alveolar hypoventilation during sleep (67, 69). Unfortunately, complications from NIPPV-induced pressure can lead to gastric distention, gastroesophageal reflux, pulmonary aspiration, and other adverse effects (67, 70). Few studies have analyzed the effectiveness of HFNO compared to other methods in children with hypercapnia. However, no studies have confirmed the effectiveness of NIV in children with acute cystic fibrosis exacerbations, and no validated criteria currently exist to determine when to initiate long-term NIV in pediatric cases (67, 69). One crossover study of hospitalized adults with cystic fibrosis found that HFNO significantly reduced RR and minute ventilation compared to NIV (71). Multiple trials are underway, which may provide critical information needed for evidence-based clinical recommendations.

### Type III respiratory failure

Post-operative respiratory failure is associated with morbidity and mortality in surgical patients. HFNO was given a conditional recommendation with moderate certainty using GRADE guidelines by a joint panel of experts within the European Society of Intensive Care Medicine for usage post-operatively in high-risk and obese patients after cardiac and thoracic surgery (9, 72). The greater portability of HFNO makes it an attractive option for patients that benefit from early mobilization.

In a multicenter, non-inferiority trial of patients after cardiothoracic surgery, HFNO was non-inferior to BiPAP with similar levels of treatment failure (21.9% BiPAP, 21.0% HFNO), reintubation rates (13.7% BiPAP, 14.0% HFNO) and ICU mortality (5.5% BiPAP, 6.8% HFNO) (22). Additionally, when diaphragm thickening fractions were analyzed as a measure of respiratory workload, BiPAP and HFNO significantly reduced respiratory workload compared to COT (73). A recent meta-analysis found when HFNO is used within 24 h post-operatively, there is a moderate likelihood that HFNO reduces reintubation rates and lessens escalation of respiratory support frequency compared to COT (72). However, the data was criticized for excluding a study of patients who underwent major abdominal surgery where a benefit from HFNO was not seen (74, 75). Recent data contradicts prior studies that found similar reintubation rates in HFNO-treated patients compared to COT (76) and no significant difference in post-operative complications (77).

Patients are also at risk for type 3 respiratory failure in the immediate post-extubation period. HFNO significantly reduced reintubation rates and post-extubation respiratory failure incidence compared to COT and performed similarly to NIPPV (78, 79). Presently, there is insufficient evidence to support HFNO as routine prophylactic post-operative care, but some patient populations may benefit and further research is needed to clarify this issue.

### Type IV respiratory failure

Type 4 respiratory failure occurs due to failure of respiratory muscles resulting from hypoperfusion in shock. The physiologic concept behind using HFNO in this setting would provide supplemental oxygen and reduce work of breathing, allowing for lower cardiac output requirements to support respiration. This may enhance the ability of the patient to resolve metabolic acidosis through typical respiratory compensation methods. Treatment focuses on supporting respiration while identifying and correcting the source of shock. Few studies examined the clinical role of HFNO during shock-induced respiratory failure. Mauri et al. (23) found that HFNO significantly reduced respiratory effort, drive, and rate in septic and septic shock patients compared to COT (23).

The authors measured respiratory effort by esophageal pressure and correlated it with plasma lactate levels and dynamic lung compliance. Both factors independently increased respiratory effort when plasma lactate levels increase, or dynamic lung compliance worsens (23). Further studies are needed to determine what, if any, impact this might have on clinical outcomes.

## Use of high flow nasal oxygen in procedures

Many patients benefit from preoxygenation prior to endotracheal intubation (80). Obese ICU patients preoxygenated with HFNO had a significantly reduced risk of severe hypoxemia compared to patients managed with a non-rebreather mask (81). In contrast, the FLORALI-2 study of 313 adult ICU patients with AHRF and preoxygenated with either NIPPV or HFNO prior to intubation found no significant difference in severe hypoxemia incidence between patients treated with NIPPV (23%) or HFNO (27%) (24).

In a recent meta-analysis, patients preoxygenated with HFNO prior to endotracheal intubation had significantly shortened ICU LOS (mean = 1.8 days). Subgroup analysis demonstrated that HFNO significantly reduced severe hypoxemia incidence during endotracheal intubation in patients with mild hypoxemia (PaO_2_/FiO_2_ > 200 mmHg), with a number needed to treat (NNT) = 16.7. The authors concluded that there was no apparent benefit to HFNO use compared to standard care for non-hypoxic patients (82).

HFNO usage during apnea has recently been studied using the newly coined “Transnasal Humidified Rapid Insufflation Ventilatory Exchange” (THRIVE) technique, where HFNO maintains oxygenation during intubation and extends apnea time (83, 84). HFNO administration reported an average apneic time of 17 min for difficult airways in 25 adult patients (85).

The THRIVE technique (HFNO with jaw support) was studied in 48 healthy children (0–10 yo) undergoing general anesthesia; results showed significantly longer apnea without desaturation times during intubation compared to jaw support alone (86). Recently, the SHINE study compared HVNI to standard care for preoxygenation of neonates undergoing endotracheal intubation. Here, 50% of first-attempt intubations were successful with HVNI compared to 31.5% with standard of care (87). Desaturation in HVNI-treated neonates occurred at a lower percentage with a longer mean time to desaturation (44.3 and 35.5 s, respectively); NNT =6 (87). These results suggest HVNI improves intubation success with lowered risk of adverse events and these data suggest that neonates, infants, and children likely benefit from preoxygenation with HFNO before intubation. Preoxygenation with HFNO is likely to benefit some patient groups and is non-inferior to NIPPV for patients with obesity (80, 82, 88).

Gastrointestinal (GI) endoscopy procedures may have complications stemming from sedation, such as respiratory depression, airway obstruction, and decreased chest wall compliance, which may induce hypoxia (89). Recent RCTs have explored using HFNO during GI procedures compared to standard methods. In the ODEPHI trial, HFNO usage during GI endoscopy reduced desaturation frequency compared to COT and significantly reduced the need for maneuvers to maintain the upper airways (25).

Evidence from other GI procedures produced similar results; patients undergoing advanced esophagogastroduodenoscopy with HFNO had an absolute risk reduction of 11.9% of hypoxic events compared to patients provided oxygen with low flow nasal cannulas (LFNC); NNT = 8.4 (90). Similarly, in a trial comparing LFNC to HFNO in patients undergoing endoscopic retrograde cholangiopancreatography (ERCP), HFNO patients had no hypoxemic events. The lowest mean SpO_2_ was higher than LFNC, suggesting that HFNO provided superior oxygenation support during ERCP (91). In a large trial of adult outpatients undergoing elective gastroscopy with propofol sedation, there was significantly lower incidence of adverse events and subclinical respiratory depression in HFNO patients. Additionally, results showed a significant reduction in mild and severe hypoxia compared to patients given LFNC (89). These data indicate that undifferentiated patients undergoing GI procedures may benefit from HFNO.

However, high-risk patient studies failed to observe any benefits. Morbidly obese (BMI >40 kg/m^2^) patients undergoing elective colonoscopy with propofol sedation were supported with HFNO or LFNO with no significant differences in desaturation incidence (92). Additionally, the OTHER trial found no significant difference in hypoxemic events in high-risk adults during ECRP (26). More high-quality studies are needed to evaluate patient populations with the highest clinical benefit.

## Conclusion

HFNO is a valuable addition to the options for managing respiratory distress. HFNO is more often portable than NIPPV, allowing greater freedom of movement for the patient and the ability to eat and speak with healthcare providers and loved ones. Additionally, HFNO patients may be managed in a range of hospital bedding areas due to mechanical constraints of NIPPV machines. Overall, more studies are needed in pediatrics, peri-operative patients, during medical procedures, type 4 respiratory distress, COVID-19, and unique patient populations.

## Author contributions

KW, NG, and JW participated in the conception, development, and writing of this manuscript. All authors agree to be accountable for the content of the work. All authors contributed to the article and approved the submitted version.

## Conflict of interest

Author JW is VP of Clinical Research for Vapotherm, Inc–a manufacturer of high flow oxygen systems. Author KW has been employed within the past 12 months as a scientific consultant. The remaining author declares that the research was conducted in the absence of any commercial or financial relationships that could be construed as a potential conflict of interest.

## Publisher's note

All claims expressed in this article are solely those of the authors and do not necessarily represent those of their affiliated organizations, or those of the publisher, the editors and the reviewers. Any product that may be evaluated in this article, or claim that may be made by its manufacturer, is not guaranteed or endorsed by the publisher.
